# Supplementary material for: 2’-Fucosyllactose Inhibits Coxsackievirus Class A Type 9 Infection by Blocking Virus Attachment and Internalisation
Source: Int J Mol Sci. 2022 Nov 8;23(22):13727. doi: 10.3390/ijms232213727 (PMC9691179; doi:10.3390/ijms232213727)
Supplement: Supplementary file 1 [file ijms-23-13727-s001.zip › ijms-1962330-supplementary.pdf]

Supplementary Materials

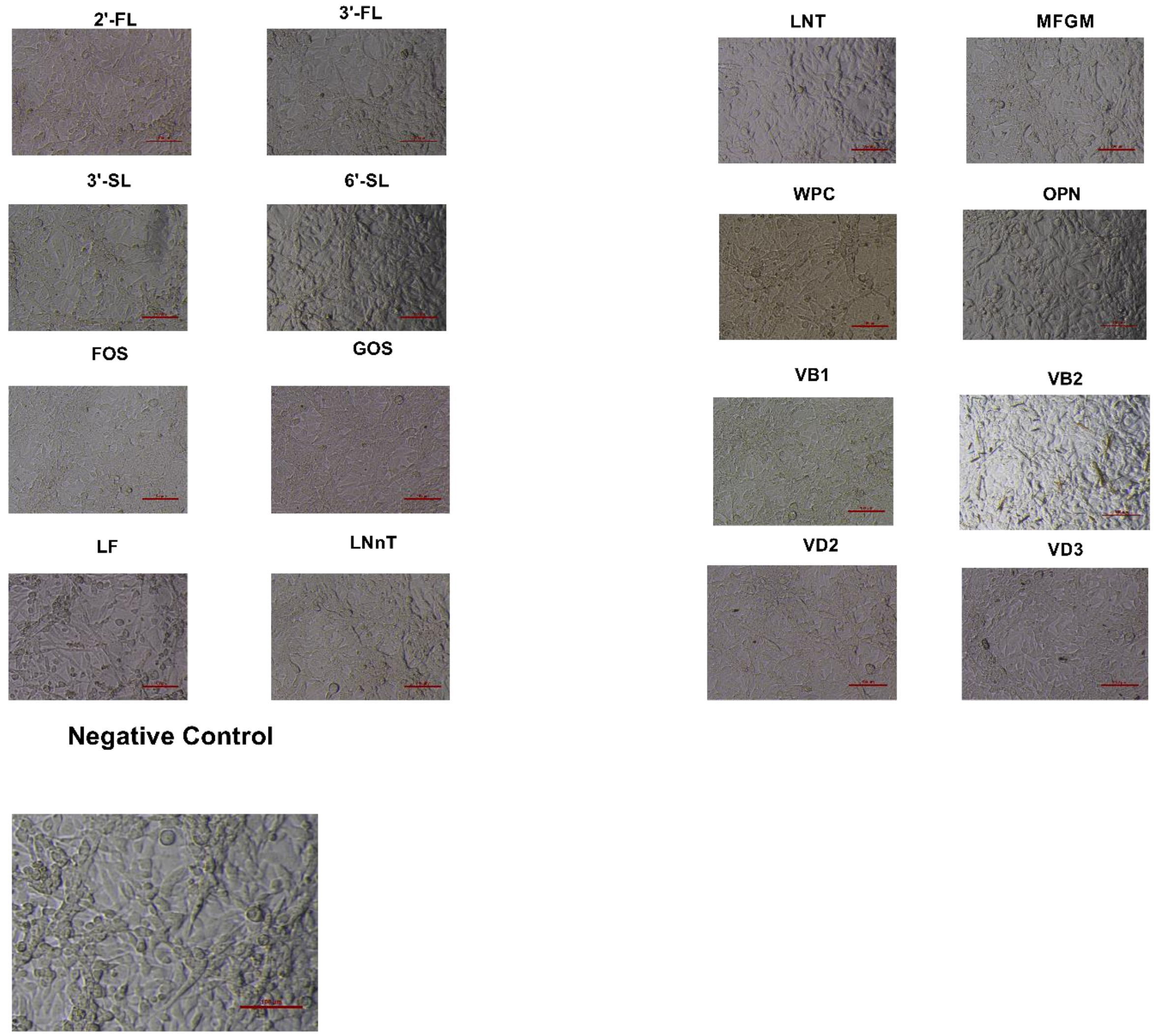

Figure S1. Cytogram after treatment with 16 primary screening drugs. RD cells were seeded in 96-well plates. At 80%–90% cell density, various drugs (2′-FL [10 mg/mL], 3′-Fucosyllactose [10 mg/mL], lacto-N-Tetraose [10 mg/mL], lacto-N-Neotetraose [10 mg/mL], 3′-Sialyllactose [10 mg/mL], 6′-Sialyllactose [10 mg/mL], whey protein concentrate [5 mg/mL], milk fat globule membrane [5 mg/mL], LF [5 mg/mL], OPN [5 mg/mL], Vitamin B2 [100 ug/mL], Vitamin B1 [1 mg/mL], Vitamin D2 [1 mg/mL], and Vitamin D3 [1 mg/mL]) were added. At 48 h.p.i., Perform cytography to observe cytopathic lesions.

A

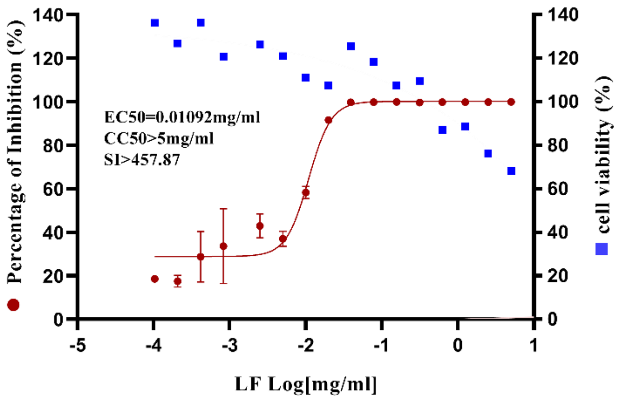

B

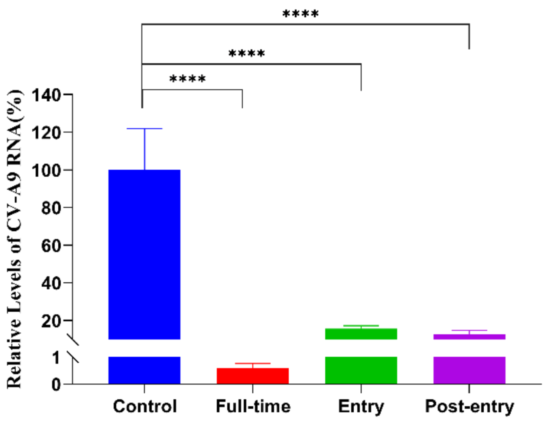

Figure S2. The anti-CV-A9 activity of lactoferrin (LF). (**A**) The anti-CV-A9 EC_{50} and CC_{50} of LF on RD. Cells were infected with CV-A9 at MOI = 0.001 and treated with LF at various concentrations (from 5mg/ml to 0.0001 mg/mL) to determine viral inhibition and cell viability. Concentration of ≥0.02 mg/mL was able to inhibit CV-A9 infection. The left and right Y-axis represent mean percentage of inhibition of virus yield and cytotoxicity, respectively. (**B**) Time-of-addition experiment of LF (0.04 mg/mL). The detailed steps for time-of-addition experiment were described in the method section, and virus and GAPDH mRNA levels in the infected cell were quantified by qRT-PCR at 48h p.i. ****$p < 0.0001$, Student's t-test compared to control.

A

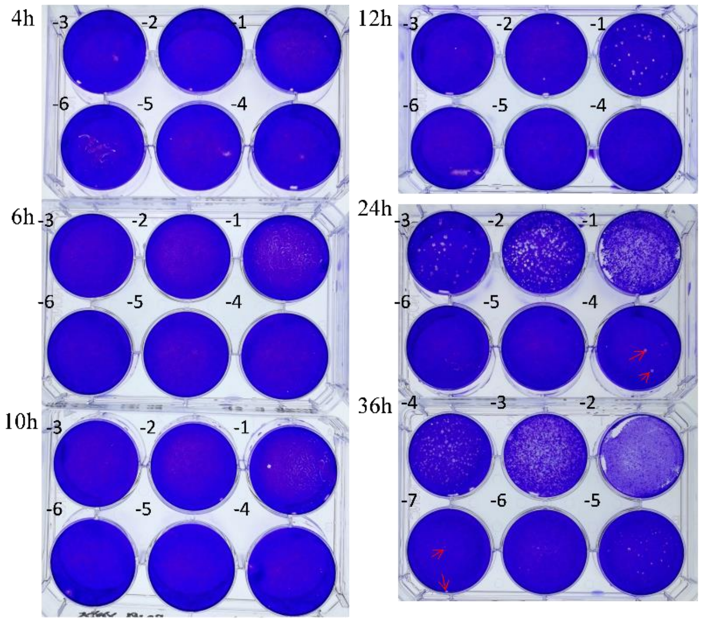
4h
-3 -2 -1
-6 -5 -4
12h
-3 -2 -1
-6 -5 -4
6h
-3 -2 -1
-6 -5 -4
24h
-3 -2 -1
-6 -5 -4
10h
-3 -2 -1
-6 -5 -4
36h
-4 -3 -2
-7 -6 -5

B

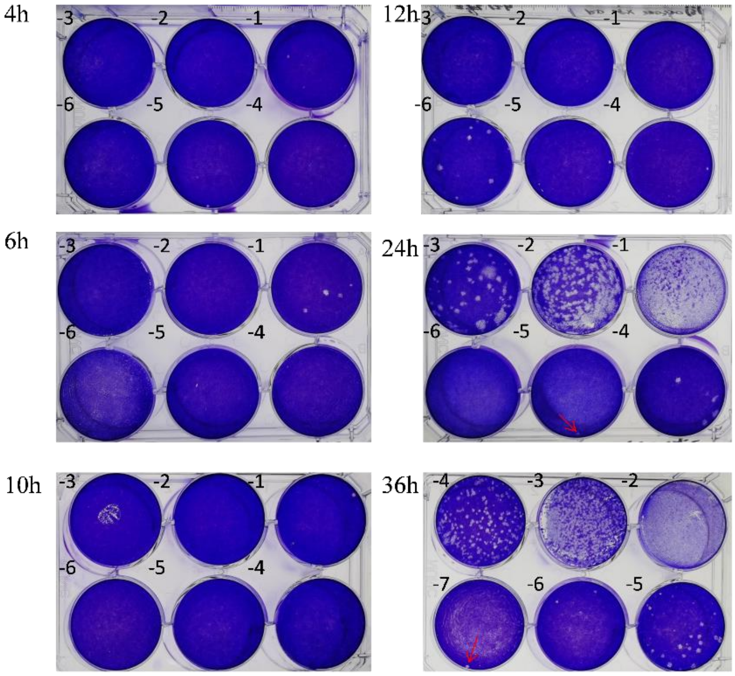
4h
-3 -2 -1
-6 -5 -4
12h
-3 -2 -1
-6 -5 -4
6h
-3 -2 -1
-6 -5 -4
24h
-3 -2 -1
-6 -5 -4
10h
-3 -2 -1
-6 -5 -4
36h
-4 -3 -2
-7 -6 -5

C

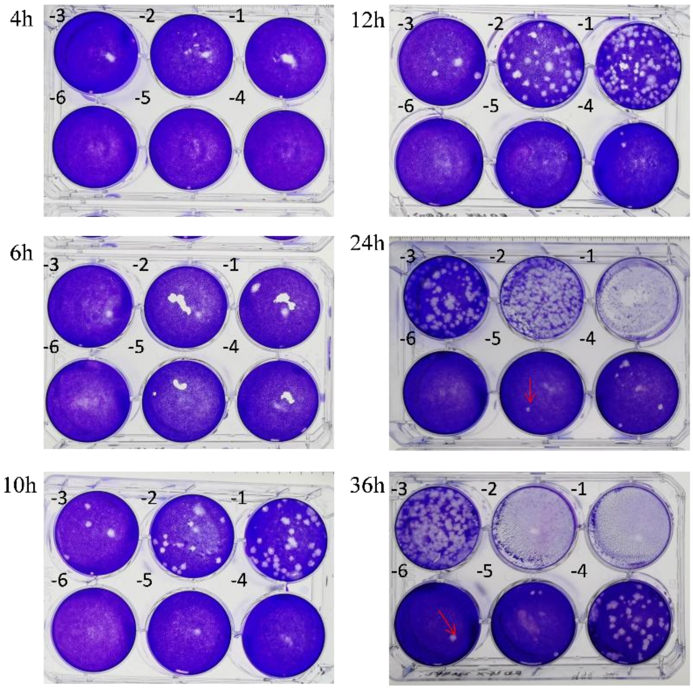

Figure S3. Plaque assay corresponding to the CV-A9 single-step growth curve in RD cells (**A**) without any drug treatment, (**B**) with a single dose of 2′FL (10 mg/ml) and (**C**) with a single dose of LF (0.04 mg/mL) treatment.
